# Excision of Three-Fourths of the Spleen

**Published:** 1875-03

**Authors:** 


EXCISION OF THREE-FOURTHS OF THE SPLEEN.—The following novel case, published by Dr. H. C. Markman (*Medical Record,*) September 15, 1874, is interesting from many standpoints. The subject was an Indian. In a quarrel his abdomen was penetrated so that three-fourths of his spleen protruded from the wound. Ere this was seen by the doctor, the projecting part of the spleen had become sphacelated. This part was removed by the knife, and the fearful hemorrhage checked by ligatures. The patient became pulseless, and a fatal issue seemed inevitable. Whisky was given, and cold water dressings applied. The patient fully recovered.—*Detroit Review of Medicine and Pharmacy.*

SIN has many tools, but a lie is the handle that fits them all. *O. W. Holmes.*

